# Fenchone, a monoterpene: Toxicity and diuretic profiling in rats

**DOI:** 10.3389/fphar.2023.1119360

**Published:** 2023-01-26

**Authors:** Asifa Bashir, Muhammad Naveed Mushtaq, Waqas Younis, Irfan Anjum

**Affiliations:** Faculty of Pharmacy, The University of Lahore, Lahore, Pakistan

**Keywords:** LD_50_, oxidative stress, fenchone, sodium, potassium

## Abstract

Fenchone is a monoterpene present in the essential oils of various plants, including *Foeniculum vulgare* and *Peumus boldus*. Previous studies confirmed the anti-inflammatory, antioxidant, wound-healing, antidiarrheal, antifungal, antinociceptive, and bronchodilator activities of fenchone. Owing to various pharmacological activities of Fenchone, the current research was designed to evaluate its diuretic activity along with toxicity profiling. For evaluating acute toxicity, OECD guideline 425 was followed in which a single dose of 2000 mg/kg was orally administered to rats. For evaluating the diuretic potential in rats, three doses of Fenchone (100, 200, and 400 mg/kg) were assayed in comparison to furosemide (15 mg/kg) as the standard drug, followed by measurements of urinary volume, urinary electrolytes, uric acid, and urinary creatinine in saline-loaded rats for 8 h. The acute toxicity study showed a significant increase in hemoglobin (Hb), red blood cells (RBCs), alkaline phosphatase (ALP), and alkaline transaminase (ALT) along with a significant decrease in serum triglycerides, cholesterol, and uric acid levels when compared with the control group. The oxidative stress parameter, superoxide dismutase (SOD), was increased in the heart and spleen. Nitrite (NO) and glutathione were significantly increased in the kidney. The acute diuretic effect of Fenchone (400 mg/kg) significantly increased the urinary output, electrolytes (Na^+^, K^+^, and Ca^++^), urinary creatinine, and urinary uric acid in a dose-dependent manner. The Na^+^/K^+^ ratio was remarkably higher in the treatment group than that of the control group. The diuretic index, saluretic index, and Lipschitz value were also calculated from electrolyte concentration and urinary volume measurements, and the values were significantly increased in rats administered with fenchone at 400 mg/kg dose. The current study concluded that fenchone is safe and has remarkable diuretic action.

## Introduction

In order to develop and test newer molecules before clinical trials, toxicity studies of compounds are fundamental (Anwar et al., 2021). A very vital consideration for the toxicity studies is ensuring the safety of the animals exposed to the study compounds (Morton, 1998). International governing organizations for toxicity determines dose-related perils on human subjects by evaluating the effect of study compounds on animals (Lewis et al., 2002). Toxicity profiling allows easy assessment of tissues along with the approximation of several biochemical and pathophysiological parameters (Dandekar et al., 2010; Arome and Chinedu, 2013). An association can be established between toxic and therapeutic doses by conducting toxicity studies of newer agents, and these extensive studies allow the researchers to determine whether to further proceed with clinical trials or not (Aneela et al., 2011). These experiments not only help in determining the safety of the newer agents in animal models but also foster the drug’s suitability for its therapeutic use (Jothy et al., 2011).

Natural therapies from medicinal plants are vital for developing new treatment protocols. Most of the current therapies have been isolated from natural plant extracts and semi-synthesized or chemically synthesized using natural constituents (Wright, 2019). Due to recent technological advances, natural therapies serve as a preliminary source of developing newer and innovative therapeutic medications, encompassing a vast range of conditions (Shen, 2015). Modern research on drug discovery from medicinal plants includes complex methods linking phytochemical, biological, botanical, and molecular activities (Balunas and Kinghorn, 2005). Phytochemicals, as a part of plant constituents, express distinct bio-activities with better acceptability, lesser side effects, better safety profile, and more cost-effective approaches. Also, they are broadly inspected for their capacity to offer better health outcomes (Krum and Pellizzer, 1998).

Diuretics are the mainstay for treating hypertension, ascites, pulmonary edema, and congestive heart failure and are either used in combination with other agents or alone (Lahlou, Tahraoui, Israili, and Lyoussi, 2007). The irrational use of diuretics poses certain restrictions regarding their side effects, including metabolic imbalances, electrolyte disturbances, activation of the renin–angiotensin aldosterone system (RAAS), and sexual dysfunction, and these side effects lead to the quest for novel diuretic agents from plant sources (Schlickmann et al., 2018).

Fenchone exists as a bicyclic monoterpene and is present in the essential oil of various fragrant plants, including *Foeniculum vulgare* and *Peumus boldus* (Kim, Song, Cho, and Lee, 2020). Previous pharmacological research has confirmed the anti-inflammatory, antioxidant, wound-healing, antidiarrheal, antifungal, antinociceptive, and bronchodilator activities of fenchone (Him, Ozbek, Turel, and Oner, 2008; Slavchev et al., 2014; Keskin et al., 2017; de Souza Pessoa et al., 2020; Rehman, Ansari, Samad, and Ahmad, 2022). The therapeutic efficacy of the fruits of *Foeniculum vulgare* is generally accredited to its essential oils. Various research studies uncovered that the essential oils as well as the individual elements demonstrate unique therapeutic potentials (Rather, Dar, Sofi, Bhat, and Qurishi, 2016). Therefore, the modern medicine era necessitates designing and developing newer diuretic agents that not only enhance the cardiovascular potentials by reducing mortality but also overcome the harmful effects of the drugs (Younis et al., 2021). Fenchone being present in many aromatic plants has shown to be a promising diuretic for treating hypertension (Agarwal, Gupta, Agrawal, Srivastava, and Saxena, 2008). Therefore, based on the reported results of the plant, fenchone was selected as the study compound and its diuretic potential and acute toxicity in rat models were determined.

## Materials and methods

### Chemicals and drugs

The chemicals employed for the research included pyrogallol solution from Oxford Labs (India); trichloroacetic acid, Griess reagent, and Ellman’s reagent (DNTB) from Omicron Sciences Limited (United Kingdom); and potassium phosphate buffer, carboxymethyl cellulose (CMC), thiobarbituric acid, furosemide, and fenchone (CAS No: 7,787-20-4) purchased from Sigma-Aldrich (United States).

### Animals

Adult Wistar albino rats (weighing 150–250 gm) of age 4–8 weeks were housed in the animal house of The University of Lahore, Pakistan. All the animals were placed under normal conditions, provided with chow and water, and kept in a well-maintained atmosphere in a 12 h light/dark cycle at proper temperature (25°C ± 1°C). All animals were provided with housing environments in accordance with accepted principles for laboratory animal use and care (NIH publication number # 85-23, revised in 1985). All experimental procedures were approved (IREC-2022-17) by the Institutional Ethical Committee of the Faculty of Pharmacy, The University of Lahore.

### Acute oral toxicity

The OECD 425 guidelines were followed for assessing acute oral toxicity. Healthy female albino rats were fasted overnight but had free access to water (OECD, 2001). Initially, fenchone was administered at 2000 mg/kg dose orally for the limit test to a single rat according to body weight, and the animal was closely observed for 30 min and then for next 4 h and 24 h. If there were no mortality rates, the same dose was administered to four other rats, and the rats were monitored for any alteration in general behavior, allergic reactions, body weight, and mortality for 14 days. The same protocol was followed for the normal control, which received only .5% carboxymethylcellulose (CMC) as a vehicle according to their body weight (Anwar et al., 2021).

### Estimation of hematological and biochemical parameters

The rats were anesthetized by administering 3%–4% isoflurane by diluting it with oxygen, and blood was withdrawn by cardiac puncture after 14 days. The hematological parameters encompassed hemoglobin (Hb), red blood cells (RBCs), packed cell volume (PCV), mean corpuscular volume (MCV), mean corpuscular hemoglobin (MCH), mean corpuscular hemoglobin concentration (MCHC), and white blood cells (WBCs), while blood was isolated in tubes having EDTA sodium, followed by centrifugation at 1,400 *g* for 10–15 min for the isolation of serum for measuring various biochemical parameters like AST, ALT, ALP, bilirubin, creatinine, uric acid, triglycerides, and cholesterol. All these parameters were assessed by utilizing their specific kits (Konan, Bacchi, Lincopan, Varela, and Varanda, 2007).

### Estimation of oxidative stress parameters

Lastly, rats were killed by cervical dislocation, and vital organs like the heart, liver, kidney, and spleen were excised and weighed separately. The tissue homogenate was prepared by mixing 1 gm of tissue with 10 mL of phosphate buffer (.1 M, pH 7.4) followed by centrifugation. The supernatant was isolated for the measurement of oxidative stress parameters like GSH, SOD, NO, and MDA (Saleem et al., 2017; Anwar et al., 2021). All the tests were executed in triplicate.

### Superoxide dismutase (SOD) estimation

A volume of 2.8 mL of potassium phosphate buffer (.1 M, pH 7.4) was mixed with .1 mL of tissue homogenate, followed by .1 mL pyrogallol solution. Mixture absorbance was measured at 325 nm, and the following SOD standard cure regression line was drawn for estimating SOD values (Nazir et al., 2021):

Y=0.0095X+0.1939.

### Nitrite (NO) level estimation

The supernatant of tissue homogenate and Griess reagent were taken in equal proportion and mixed thoroughly, and this mixture was allowed to incubate for 10 min, followed by the measurement of absorbance at 546 nm. A sodium nitrite regression line was used for estimating nitrite levels (Nazir et al., 2021) as follows:

Y=0.003432X+0.0366.

### Glutathione (GSH) estimation

Tissue homogenate (1 mL) was precipitated with 10% trichloroacetic acid (1 mL) along with phosphate buffer (4 mL). The aliquot supernatant was separated after precipitation followed by the addition of .5 mL of 5, 5 dithiobis 2 nitrobenzoic acid (DNTB). Mixture absorbance was measured at 412 nm, and the following formula was utilized for estimating the amount of GSH in the tissue homogenate (Saeed, Saleem, Anwar, Ahmad, and Anwar, 2020):

$$\mathrm{Glutathione}\left(GSH\right)=Y-\frac{0.00314}{0.034}\times \frac{DF}{BT}\times VU,$$

where Y is the absorbance at 412 nm, DF is the dilution factor, BT is the tissue homogenate, and VU is the volume of aliquot.

### Malondialdehyde (MDA) estimation

Tissue homogenate (1 mL) was added to 3 mL of thiobarbituric acid (TBA) reagent. The resulting mixture was stirred and allowed to incubate for 15 min at room temperature, followed by cooling on an ice bath and centrifugation. The layer of supernatant was separated for measuring the absorbance at 532 nm. The following formula was utilized for estimating the concentration of MDA in tissue homogenate (Nazir et al., 2021):

$$Conc\;of\;MDA=\frac{Abs\;532\times 100\times V_{t}\;}{1.56\times 10^{5}\times W_{t}\times V_{u}\;},$$

where V_t_ is the total volume mixture, 1.56×10^5^ is the extinction coefficient, W_t_ is the weight of the dissected tissue, and V_u_ is the volume of aliquot.

### Histopathological evaluation

The excised organs including the heart, liver, kidney, and spleen were placed in paraformaldehyde (4%), fixed in paraffin, and cut into 5-µm sections, followed by staining with eosin and hematoxylin and observed using the microscope for any pathological changes (Anwar et al., 2021).

### Assessment of acute diuretic activity of fenchone

#### Acute diuretic activity

The rats were placed in metabolic cages 7 days prior to the procedure to acclimatize them to the experimental conditions. The rats, weighing 150–250 gm, were divided into five groups, with five rats in each group. Initially, all the rats received 5 mL/100 gm (p.o) of .9% normal saline to ensure a uniform water and salt load (Younis et al., 2021). After 45 min, Group I, which was the normal control group, received only .5% CMC (1 mL/kg), while Group II was the reference standard and received 15 mg/kg furosemide by the oral route, and the test groups (III, IV, and V) were given 100, 200, and 400 mg/kg of fenchone, respectively, through oral gavage.

Animals were kept in metabolic cages, and urine sampling was carried out after 2, 4, and 6 h of treatment for analysis of Na^+^, K^+^, Ca^++^, urinary creatinine, pH, and uric acid.

#### Electrolyte estimation

pH was measured using a pH meter, and Na^+^, K^+^, Ca^++^, uric acid, and urinary creatinine were measured (Prathibhakumari and Prasad, 2014) using Bio-active Diagnostic System kits.

#### Diuretic index, saluretic index, and Lipschitz value

Based on the urine volume and electrolyte concentrations, the diuretic index, saluretic index, and Lipschitz value were also calculated.

Diuretic index = urinary volume of the treatment group/urinary volume of the normal control group,

Saluretic index Na^+^(SI_Na_^+^) = urinary Na^+^ concentration of the treatment group/urinary Na^+^ concentration of the normal control group,

Saluretic index K^+^(SI_K_^+^) = urinary K^+^ concentration of the treatment group/urinary K^+^ concentration of the normal control group,
Saluretic index (SI) = SI_Na_^+^ + SI_K_^+^ of the treatment group/SI_Na_^+^ + SI_K_^+^ of the normal control group,
Lipschitz value = urinary volume of the treatment group/urinary volume of the standard group.

### Statistical analysis

The results of the experiments were evaluated using GraphPad Prism version 5, and the data were presented as mean ± SEM. Both one-way ANOVA and two-way ANOVA were used for analyzing the data statistically, followed by the Tukey comparison test and Bonferroni and Dunnett’s *post hoc* tests. *p*< .05 was considered significant. *p* < .01 and *p* < .001 indicated moderate and highly significant levels, respectively.

## Results

### Effect of treatment on the behavioral pattern in the acute toxicity study

Table 1 shows that no mortality or morbidity occurred during the acute toxicity study with fenchone (F) treatment (2000 mg/kg). No significant difference was noted in behavioral parameters when compared to the normal control group.

**TABLE 1:** Behavioral changes after treatment with a single dose of fenchone (F) (2000 mg/kg) in the acute oral toxicity study when compared to those of the normal control.

Parameter	Normal control (NC)	Treatment group
Fur and skin	N	N
Eyes	N	N
Salivation	N	N
Respiration	N	N
Urination (color)	N	N
Feces consistency	N	N
Somatomotor activity and behavior pattern	N	N
Sleep	N	N
Mucous membrane	N	N
Convulsions and tremors	NF	NF
Itching	NF	P
Coma	NF	NF
Mortality	NF	NF

N, normal; NF, not found; P, present.

### Effects of treatment on body weight and organ weight

The body weight of the treated animals was noted from the first day to the 14th day. The treatment group showed a parallel increase in body weight similar to the normal control group in the early days, but a significant decrease was observed on the 14th day in comparison with the first day as shown in Tables 2, 3. A non-significant difference was observed in all organ weights when compared with the control group.

**TABLE 2:** Effects of treatment (F) on body weight of rats in the acute toxicity study.

Body weight	Normal control (NC)	Treatment group
0 day body weight (gm)	168 ± 7.90	165.2 ± 8.3
First day body weight (gm)	169 ± 7.90	166.5 ± 8.3
Seventh day body weight (gm)	169.5 ± 7.70	161.2 ± 11.3
Fourteenth day body weight (gm)	168.5 ± 6.61	161.5 ± 11.9*

Values are shown as mean ± SEM; N = 6, *p < .05 in comparison with the first day.

**TABLE 3:** Effects of F (2000 mg/kg) dose on organ weight in the acute toxicity study.

Organ	Normal control (gm)	Fenchone (2000 mg/kg)
Heart	.52 ± .021	.52 ± .035
Kidney	1.74 ± .02	1.71 ± .014
Liver	7.03 ± .02	7.02 ± .018
Spleen	.53 ± .02	.50 ± .028

Values are presented as mean ± SEM, N = 6.

### Treatment effect on complete blood count in the acute toxicity study

On the 14th day, blood was withdrawn by cardiac puncture from the treated animals. A significant rise in the RBC count and Hb level was observed in animals treated with fenchone (2000 mg/kg) when compared with those of the control group as shown in Table 4. However, other parameters are parallel to the normal control group.

**TABLE 4:** Analysis of complete blood count in animals treated with fenchone (2000 mg/kg) in the acute toxicity study.

Parameter	Unit	Normal control (NC)	Fenchone (2000 mg/kg)
Hb	G/dL	10.74 ± .01	15.2 ± .15*
RBCs	×10^6^/µL	3.64 ± .02	8.1 ± .05*
PCV	%	43.0 ± .57	41 ± .57
MCV	f/L	101.66 ± .38	52.6 ± 1.45
MCH	pg/L	25.52 ± .27	19.7 ± .23
MCHC	G/dL	25.35 ± 0.1	37.6 ± .63
WBCs	×10^3^/µL	7.3 ± .10	7.0 ± .05

Values are presented as mean ± SEM, N = 6. *p < .05 in comparison with the control group.

### Analysis of biochemical markers in treatment groups in the acute toxicity study

Table 5 illustrates that animals treated with fenchone (2,000 mg/kg) showed a significant decrease in the triglyceride and cholesterol levels in comparison with those of the control group. However, hepatic functioning markers were significantly higher than those of the control group. Surprisingly, fenchone significantly decreased the uric acid levels in the treated animals when compared with the control.

**TABLE 5:** Effect of treatment on biochemical parameters.

Biochemical marker		Unit	Normal control	Fenchone (2000 mg/kg)
Lipid profile	Triglycerides	mg/dL	197 ± 1.15	135 ± 1.15**
	Cholesterol	mg/dL	223 ± 1.45	192 ± 1.152**
	HDL	mg/dL	22.5 ± .65	27.5 ± 1.23
Renal function test	Creatinine	mg/dL	23.7 ± .176	22.4 ± .127
	Uric acid	mg/dL	23.5 ± .213	.379 ± .001*
Liver function test	ALP	U/L	186 ± 1.87	192 ± 1.9
	ALT	U/L	45 ± .34	58 ± .63*
	AST	U/L	72 ± 1.42	81 ± 2.1*
	Bilirubin	mg/dL	.95 ± .032	1.310 ± .017

Data are presented as mean ± SEM, N = 6; *p < .05, **p < .01, and ***p < .001 vs. normal control.

### Oxidative stress marker analysis in the acute toxicity study

**FIGURE 1:**
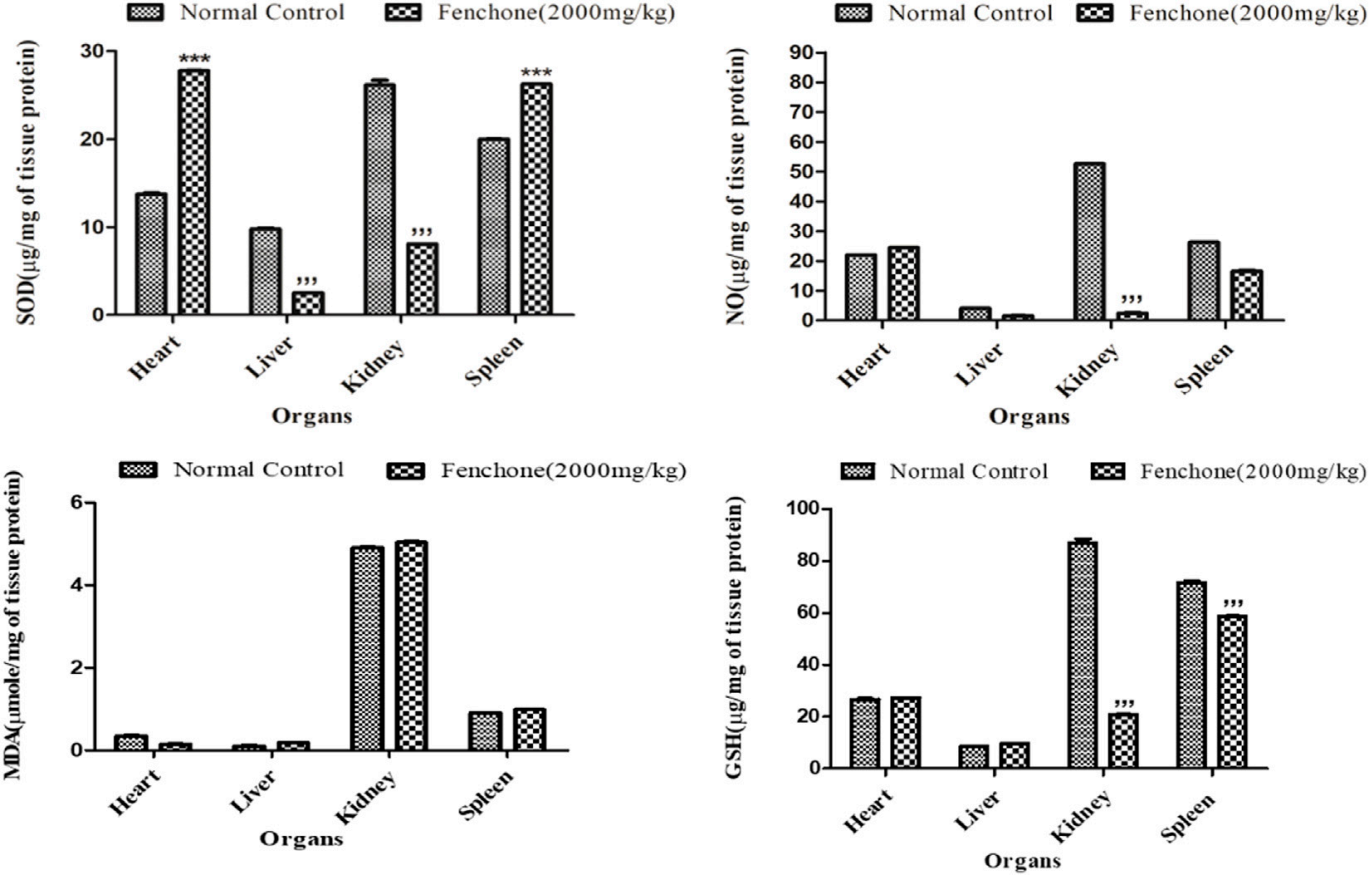
Oxidative stress marker analysis in treated animals in the acute toxicity study. Data are stated as mean ± SEM, N = 3. ***p < .001 represents a significant increase, while **’’’** *p* < .001 represents a significant decrease vs. normal control.

Figure 1 shows the results of oxidative stress biomarker analysis in the chosen organs of the treated animals. The results showed that in the kidneys of the Fenchone-treated animals, there was a significant decrease in SOD, NO, and GSH when compared to those of the normal control group. A highly significant increase in SOD levels was observed in the heart. Lipid peroxidation did not show any significant difference in all chosen organs when compared to that of the normal control group.

### Histopathological analysis

**FIGURE 2:**
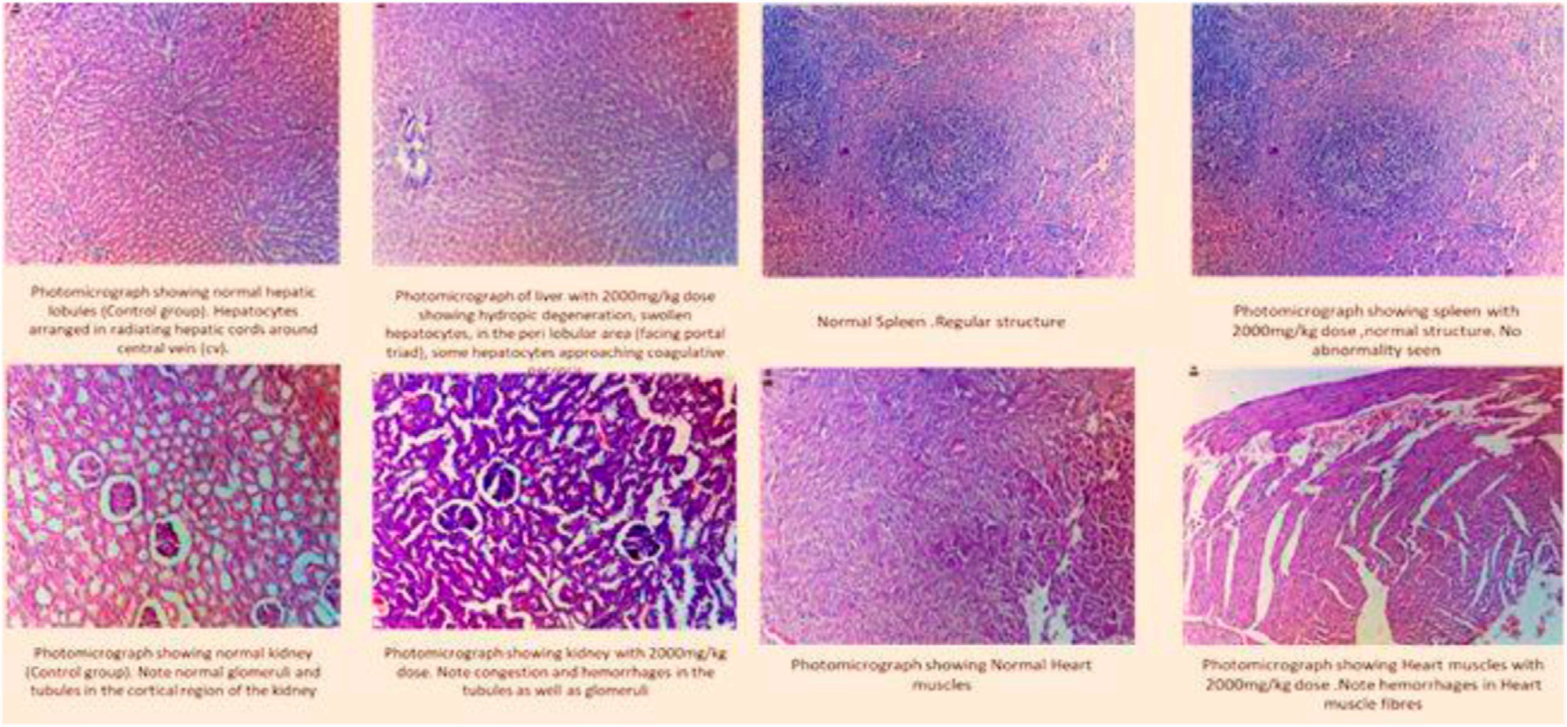
Histogram of the selected organs treated with fenchone (2000 mg/kg) in the acute toxicity study.

Histopathological analysis showed no remarkable change in the architecture of cells (Figure 2).

### Effect of different doses of fenchone on urinary electrolytes (Na^+^ and K^+^) and Na^+^/K^+^ ratio in acute diuretic activity

**FIGURE 3:**
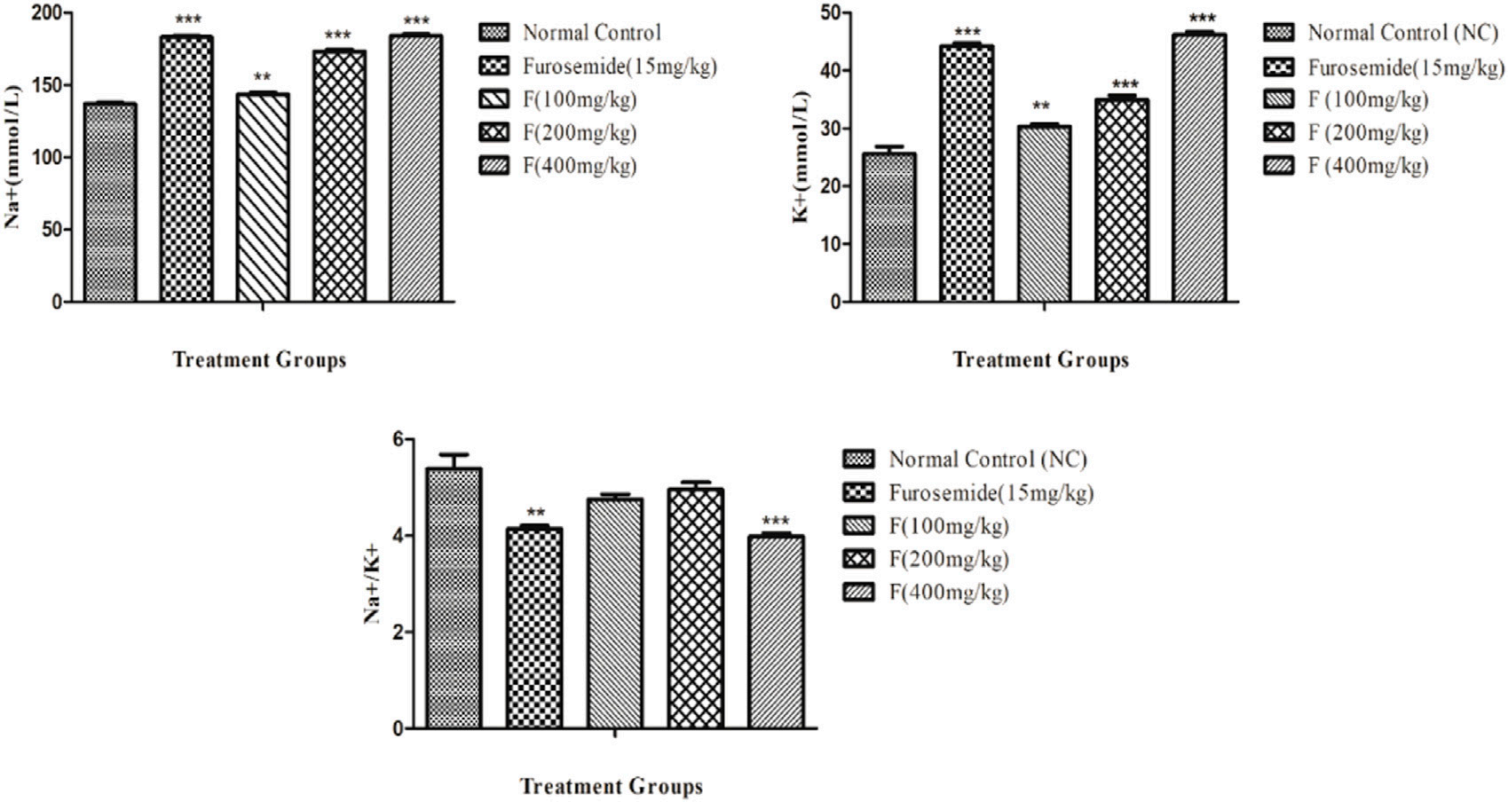
Effect of different doses of fenchone on urinary electrolytes (Na^+^ and K^+^) where furosemide was taken as the standard drug. Data are stated as mean ± SEM, N = 6. ***p < .001 represents a significant increase, while **’’’** *p* < .001 represents a significant decrease vs. normal control.

The effects of different doses of fenchone (100, 200, and 400 mg/kg) and furosemide (15 mg/kg) on sodium and potassium are shown in Figure 3. Fenchone (200 mg/kg and 400 mg/kg) resulted in a significant increase in sodium and potassium excretion. Na^+^ and K^+^ excretion rates induced by fenchone (400 mg/kg) were 184.06 ± 1.21 and 46.21 ± .51, whereas in the normal control group the rates were 136.93 ± .95 and 25.53 ± 1.31 mmol/L, respectively. Moreover, fenchone (400 mg/kg) resulted in more electrolyte excretion of sodium and potassium than furosemide. Moreover, there was a significant increase in the Na^+^/K^+^ ratio at a dose of 400 mg/kg fenchone when compared with that of the control group.

### Effect of different doses of fenchone on urinary creatinine and uric acid

**FIGURE 4:**
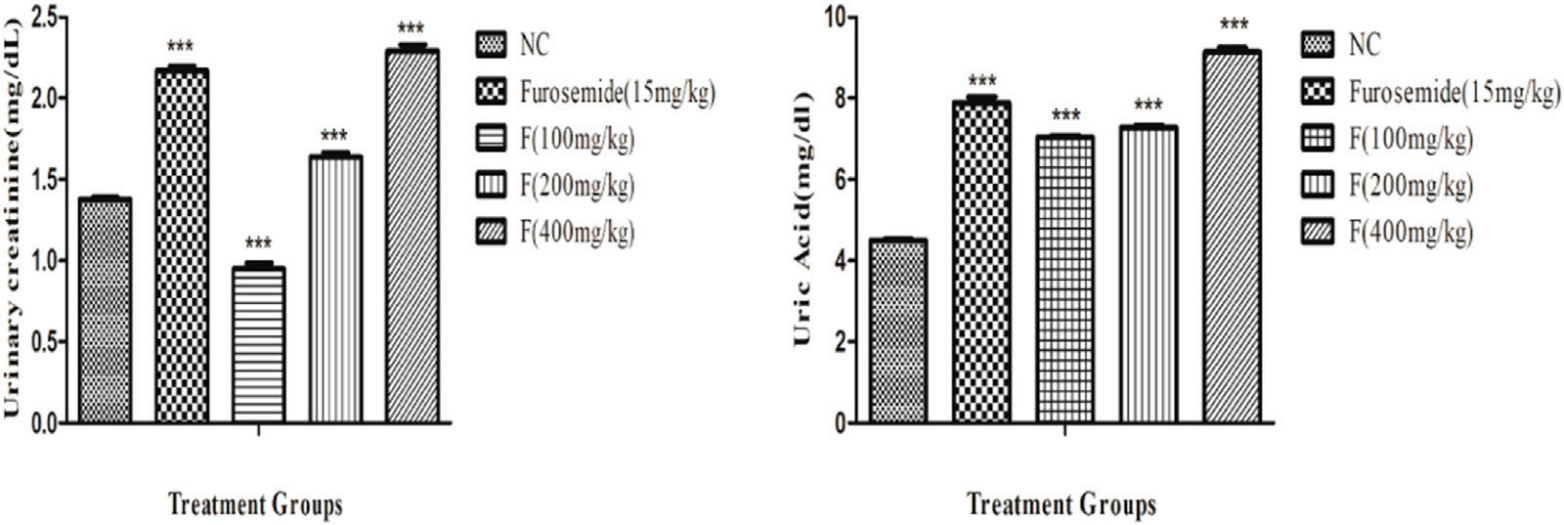
Effect of different doses of fenchone on urinary creatinine and uric acid excretion where furosemide was taken as the standard drug. Data are stated as mean ± SEM, N = 6. ***p < .001 represents a significant increase, while **’’’** *p* < .001 represents a significant decrease vs. normal control.

The effects of different doses of fenchone (100, 200, and 400 mg/kg) and furosemide (15 mg/kg) on urinary creatinine and uric acid are shown in Figure 4. There was a significant increase in the excretion of creatinine in the urine, and it was the highest with fenchone (400 mg/kg) when compared to the control. Moreover, the concentration of uric acid in the urine also increased significantly with a higher dose of fenchone (400 mg/kg), which was 9.14 ± .10 when compared to that of the normal control group, which was 4.49 ± .02. Both urinary creatinine and uric acid excretion results were comparable to furosemide.

### Effect of different doses of fenchone on urinary calcium

**FIGURE 5:**
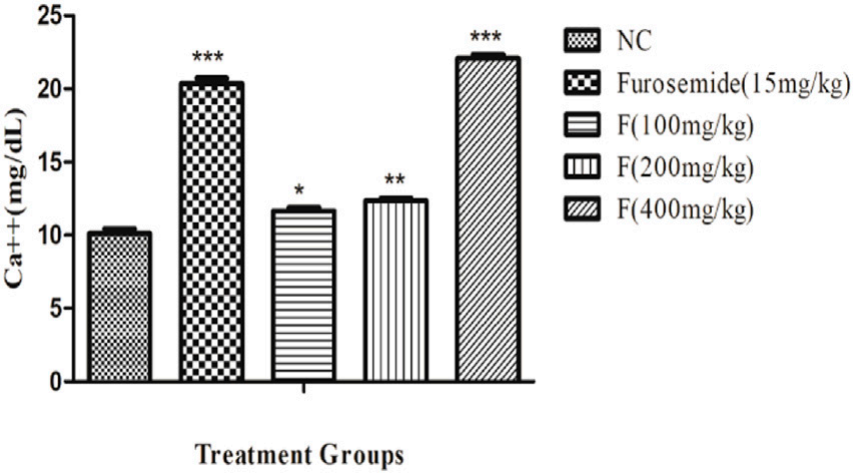
Effect of different doses of fenchone on urinary calcium where furosemide was taken as the standard drug. Data are stated as mean ± SEM, N = 6. ***p < .001 represents a significant increase, while **’’’** *p* < .001 represents a significant decrease vs. normal control.

There was a significant increase in the urinary calcium excretion, and it was the highest with fenchone (400 mg/kg) when compared to the control. The urinary excretion of Ca^++^ with fenchone at 400 mg/kg was 22.08 ± .28 when compared to that of the normal control group which was 10.11 ± .33 as shown in Figure 5.

### Effect of different doses of fenchone on urinary pH and urinary volume

The acute diuretic effect of different doses of fenchone (100, 200, and 400 mg/kg) is shown in Table 6. The fenchone (400 mg/kg) dose resulted in increased urinary output at a 6-h interval following treatment. There was a dose-dependent increase in urinary volume measured at 6 h in 400 mg/kg fenchone-treated rats which was 5.22 ± .20 mL/100 gm, while the urinary volume was 2.23 ± .14 in the control. Moreover, the urinary output at 6 h in rats treated with fenchone (400 mg/kg) was comparable to the output in rats treated with furosemide, a standard diuretic drug. Urinary output data also suggested that the increase in urine load was due to the increased polarity resulting from this activity. Finally, the urine pH values showed a significant increase with fenchone (200 and 400 mg/kg) treatment when compared with those of the normal control group.

**TABLE 6:** Effect of different doses of fenchone on pH and urinary volume.

Treatment group	pH	Urine volume mL/100 gm at 8 h
Normal control (NC)	7.17 ± .04	2.23 ± .14
Furosemide (15 mg/kg)	7.76 ± .08***	4.83 ± .29 ***
F (100 mg/kg)	7.69 ± .09 *	2.93 ± .08
F (200 mg/kg)	7.83 ± .03 ***	3.44 ± .18 **
F (400 mg/kg)	7.88 ± .05 ***	5.22 ± .20 ***

Results are stated as mean ± SEM, where *p < .05, **p < .01, and ***p < .001, when compared to the normal control group. NC, normal control group; Na^+^/K^+^, urinary concentration of sodium/urinary concentration of potassium.

### Effect of fenchone on the diuretic index, Lipschitz value, saluretic index of Na^+^ and K^+^, and saluretic index

By utilizing the excreted concentration of electrolytes (Na^+^ and K^+^) and urinary output in the control, standard, and treatment groups, the diuretic index, Lipschitz value, and saluretic index were calculated as shown in Table 7, and the results were quite significant at a dose of 400 mg/kg F.

**TABLE 7:** Effect of fenchone on the diuretic index, Lipschitz value, saluretic index of Na^+^ and K^+^, and saluretic index.

Treatment group	Diuretic index (DI)	Lipschitz value (LV)	Saluretic index (SI)		Saluretic index (SI)
			SI_Na_^+^	SI_K_^+^	
Normal control (NC)	1.00	.46 ± .04	.97 ± .01	1.00	1.00
Furosemide (15 mg/kg)	2.18 ± .23 ***	1.00***	1.33 ± .02***	1.74 ± .11***	1.40
F (100 mg/kg)	1.32 ± .12	.60 ± .03	1.04 ± .01	1.19 ± .07	1.07
F (200 mg/kg)	1.54 ± .06	.71 ± .04*	1.25 ± .01***	1.37 ± .10*	1.28
F (400 mg/kg)	2.18 ± .19 ***	1.1 ± .11***	1.33 ± .01***	1.81 ± .10***	1.41

Results are stated as mean ± SEM, where *p < .05, **p < .01, and ***p < .001, when compared to the normal control group.

## Discussion

Toxicity profiling of newer molecules has gained attention and interest since it helps provide future directions to researchers, manufacturers, and distributors. The rules and regulations for toxicity studies of newer compounds help in regulating and ensuring their safety for further use in human beings. The procedure involves administration of the test compound followed by examination and investigation of biochemical parameters in animal models (Rand and Petrocelli, 1985), since the main purpose of the toxicity study is to administer the single highest dose that could result in inducing toxic effects (Klaassen, 2013).

The acute toxicity study involved the oral administration of a single dose of fenchone (2000 mg/kg) to female rats since female rats are more responsive to any biochemical change than male rats (OECD, 2001). Until the 14th day, no mortality or fatal toxicity was noted, indicating that the LD_50_ of F is significantly greater than 2000 mg/kg. Generally, it has been observed that variations in the behavior pattern and body weight are the primary indicators of toxicity (Variya, Bakrania, Madan, and Patel, 2019), and the results indicated no change in the behavioral pattern of the animals. However, there was a significant decrease in the body weight on day 14 when compared to that of the normal control group, whereas a non-significant difference was observed in the organ weights. Fenchone, an important constituent of fennel seeds, causes weight loss by boosting metabolism (Yamini, Sefidkon, and Pourmortazavi, 2002), so the decrease in weight loss due to fenchone treatment might be attributed to this.

Both biochemical evaluation and hematological evaluation provide a genuine overview regarding toxicity along with organ dysfunction. Pluripotent stem cells give rise to white blood cells (WBCs), red blood cells (RBCs), and platelets after maturation and differentiation (Ugwah-Oguejiofor et al., 2019). The Hb level and RBC count were observed to be increased in the acute toxicity study in contrast to the control group owing to the antioxidant properties that helped in stabilizing the RBC membrane and raising the level of RBCs by influencing the kidneys and liver, thereby increasing the level of erythropoietin (Mansouri et al., 2015). All the remaining parameters were normal when compared to those of the control group.

Dyslipidemia is linked with obesity since it may cause insulin resistance in the periphery by increasing the hepatic output of fats from the diet and raising triglyceride hydrolysis (Klop, Elte, and Castro Cabezas, 2013). Triglyceride and cholesterol levels were significantly reduced in the acute toxicity study that might be attributed to altered expression of the leptin receptor (Zakernezhad et al., 2021).

Estimating biochemical parameters in the liver and kidney is of primary importance since both these organs play a vital role in drug metabolism and excretion, respectively (Bariweni, Yibala, and Ozolua, 2018), and any variation in either liver or kidney function tests shows toxicity (Abdelkader et al., 2020). Liver enzymes including ALT and AST were increased at a dose of 2000 mg/kg F that might have resulted from cell damage to the liver since ALT and AST are the enzymes present in the cytosol and mitochondria of hepatocytes (Samadi-Noshahr, Hadjzadeh, Moradi-Marjaneh, and Khajavi-Rad, 2021), whereas there was a significant decrease in the uric acid levels.

Uninterrupted exposure or formation of free oxidative radicals in living organisms can lead to disturbances in redox homeostasis, resulting in damage to cellular nucleic acids, proteins, and lipids (Jones, 2006; Zhang et al., 2020). If the interrupting stimulus is not removed, irreversible cell damage will ensue, resulting in responses of numerous defensive cascades including gene regulatory cycle, repair, and various antioxidant routes to protect against oxidative stress (Nair et al., 2015). Endogenous oxidants and antioxidants were measured in the specific organs of rats to assess any change or cellular stress induced by F dosing. The results showed that there was a significant increase in SOD levels in the heart and spleen that might be attributed to the immunomodulatory effect of the drug (Anwar et al., 2021), whereas a significant decrease was observed in the liver and kidney. Similarly, there was a significant decrease in NO and GSH levels in the kidney showing that it might have been excreted from the kidney, indicating a decline in oxidative stress markers, and these results can be correlated to the histopathological slides.

It has been established that diuretics are the mainstay for treating hypertension and edematous conditions, which are expressed as excessive extracellular fluid. In general, diuretics are known to increase the excessive salt and water excretion from the body, thus reducing blood flow resistance by decreasing blood pressure and volume (Younis et al., 2021). Even though diuretics are available abundantly for human use, the side effects pose a need for newer agents from plants or their constituents (Wright, Van-Buren, Kroner, and Koning, 2007), displaying numerous pharmacological effects on renal physiology and acting on distinct and well-established targets like nitric oxide-cGMP, carbonic anhydrase, renin–angiotensin system, and renal carriers (Aparecida Livero, Vergutz Menetrier, Luiz Botelho Lourenco, and Gasparotto Junior, 2017). In the present research, fenchone resulted in a significant dose-dependent increase in diuretic activity, with a maximum response at 400 mg/kg dose. A single dose of fenchone significantly increased the urinary output from the second hour and increased with time, and the effect was the highest with F 400 mg/kg (5.22 ± .20) after 8 h. This effect was significantly higher than that of the standard drug furosemide (15 mg/kg) which was 4.83 ± .29, but the onset time was less with fenchone. The time difference in the onset of diuretic action can be related to the absorption profiles of fenchone. The increase in the urinary output of F can be attributed to the increase in compound polarity that might have increased the renal circulation along with glomerular filtration and thus increased the formation of urine (Chen et al., 2014).

The current study also revealed that F 400 mg/kg dose also resulted in inducing a significant increase in urinary electrolytes Na^+^ (184.06 ± 1.21) and K^+^ (46.21 ± .51) when compared to those of furosemide which were 183.06 ± 1.24 for Na^+^ and 44.23 ± .49 for K^+^, showing both saluretic and kaliuretic activities that might be due to their interaction with polar groups (Martín-Herrera, Abdala, Benjumea, and Pérez-Paz, 2007). Similarly, there was an increase in the Na^+^/K^+^ ratio at F 400 mg/kg dose that might be attributed to an increase in sodium loss along with potassium retention (Feng et al., 2013), which is an essential feature of a good diuretic that produces reduced hyperkalemic effects (Ghelani, Chapala, and Jadav, 2016).

Creatinine is a reliable marker for assessing kidney function (Prathibhakumari and Prasad, 2014). Urinary creatinine was significantly increased at both 200 mg/kg and 400 mg/kg doses, but a higher effect was observed with fenchone (400 mg/kg) in a dose-dependent manner, so its use can be considered safe in patients with renal insufficiencies.

Uric acid is the end-product of nucleic acid metabolism in the human body, and this homeostasis is attained by either modulating the formation, breakdown, or excretion of uric acid. Hyperuricemia is generally characterized by an elevation in the levels of uric acid and is considered a risk factor for causing cardiovascular disease, diabetes, hypertension, chronic kidney disease, and gout (Zhang et al., 2018). The current research revealed that the F (200 and 400 mg/kg) dose resulted in a significant increase in the excretion of uric acid in the urine with much higher results at 400 mg/kg dose, and thus it can be used in treating gout.

## Conclusion

The current study showed that the LD_50_ of fenchone is greater than 2,000 mg/kg, no mortality or signs of toxicity were reported, and it has a remarkable diuretic potential comparable to furosemide. Moreover, oral treatment with F resulted in increased excretion of sodium and potassium in the urine, along with increases in the Na^+^/K^+^ ratio, diuretic index, Lipschitz value, and saluretic index. The results suggest that fenchone (F) may be considered a safe therapeutic agent in patients with cardiovascular and renal insufficiencies, but further mechanistic studies would be essential before introducing it into clinical trials.

## Data Availability

The raw data supporting the conclusion of this article will be made available by the authors, without undue reservation.
